# Characterization of the Autocrine/Paracrine Function of Vitamin D in Human Gingival Fibroblasts and Periodontal Ligament Cells

**DOI:** 10.1371/journal.pone.0039878

**Published:** 2012-06-25

**Authors:** Kaining Liu, Huanxin Meng, Jianxia Hou

**Affiliations:** Department of Periodontology, Peking University School and Hospital of Stomatology, Beijing, China; University of Chicago, United States of America

## Abstract

**Background:**

We previously demonstrated that 25-hydroxyvitamin D_3_, the precursor of 1α,25-dihydroxyvitamin D_3_, is abundant around periodontal soft tissues. Here we investigate whether 25-hydroxyvitamin D_3_ is converted to 1α,25-dihydroxyvitamin D_3_ in periodontal soft tissue cells and explore the possibility of an autocrine/paracrine function of 1α,25-dihydroxyvitamin D_3_ in periodontal soft tissue cells.

**Methodology/Principal Findings:**

We established primary cultures of human gingival fibroblasts and human periodontal ligament cells from 5 individual donors. We demonstrated that 1α-hydroxylase was expressed in human gingival fibroblasts and periodontal ligament cells, as was cubilin. After incubation with the 1α-hydroxylase substrate 25-hydroxyvitamin D_3_, human gingival fibroblasts and periodontal ligament cells generated detectable 1α,25-dihydroxyvitamin D_3_ that resulted in an up-regulation of CYP24A1 and RANKL mRNA. A specific knockdown of 1α-hydroxylase in human gingival fibroblasts and periodontal ligament cells using siRNA resulted in a significant reduction in both 1α,25-dihydroxyvitamin D_3_ production and mRNA expression of CYP24A1 and RANKL. The classical renal regulators of 1α-hydroxylase (parathyroid hormone, calcium and 1α,25-dihydroxyvitamin D_3_) and *Porphyromonas gingivalis* lipopolysaccharide did not influence 1α-hydroxylase expression significantly, however, interleukin-1β and sodium butyrate strongly induced 1α-hydroxylase expression in human gingival fibroblasts and periodontal ligament cells.

**Conclusions/Significance:**

In this study, the expression, activity and functionality of 1α-hydroxylase were detected in human gingival fibroblasts and periodontal ligament cells, raising the possibility that vitamin D acts in an autocrine/paracrine manner in these cells.

## Introduction

Vitamin D_3_ is a major component in the regulation of calcium and phosphorus metabolism. The function of vitamin D_3_, via the active hormonal metabolite 1α,25-dihydroxyvitamin D_3_ (1,25OH_2_D_3_), is to regulate the absorption of these essential minerals in the intestine, and their mobilization in bone tissues [1]. 1,25OH_2_D_3_ also plays an important role in the regulation of T cell function and immunological reaction [2]. The biological function of 1,25OH_2_D_3_ is orchestrated by vitamin D receptor (VDR). After binding with VDR, 1,25OH_2_D_3_ acts on the vitamin D responsive element (VDRE) located upstream of its target genes (eg. CYP24A1, RANKL, et al.) and up-regulates their expression which results in the corresponding biological effects [3], [4], [5], [6], [7].

1,25OH_2_D_3_ is formed from vitamin D_3_ in a two-step hydroxylation: 25-hydroxyvitamin D_3_ (25OHD_3_) hydroxylated in the liver is transported to the kidney by vitamin D binding protein (DBP) and is metabolized to 1,25OH_2_D_3_ by the renal cytochrome P450 enzyme 25OHD_3_-1α-hydroxylase CYP27B1 [8].

Besides the kidney [9], [10], [11], there are extra-renal sites of 1,25OH_2_D_3_ synthesis, including the skin [12], prostate [13], activated monocytes/macrophages [14], bone [15], [16] and sebocytes [17]. Furthermore, local production of 1,25OH_2_D_3_ in extra-renal tissues has been postulated to regulate parameters of cell growth and differentiation in an autocrine or paracrine fashion [15], [18].

Periodontal tissues consist of two hard tissues, bone and cementum, and two soft tissues, gingiva and periodontal ligament. Although bone-derived cells, osteoblast-like cells and osteoblasts can synthesize 1,25OH_2_D_3_ [15], [16], it is unknown whether this also occurs in periodontal soft tissues. Human gingival fibroblasts (hGF) and human periodontal ligament cells (hPDLC) are two kinds of periodontal fibroblasts and are important components of gingiva and periodontal ligament, respectively. Whether 1,25OH_2_D_3_ synthesis occurs in these cells warrants further investigation.

Our previous studies [19], [20] demonstrated that levels of plasma 25OHD_3_ of patients with aggressive periodontitis were significantly higher than those of healthy controls; local 25OHD_3_ levels in gingival crevicular fluids were 300 times higher than plasma levels in these patients. It is not known why the precursor of 1,25OH_2_D_3_ is abundant around periodontal soft tissues. Recently, McMahon et al. reported that 1,25OH_2_D_3_ could enhance the antibacterial defense of human gingival epithelial cells [21]. Thus, if 25OHD_3_ is converted to 1,25OH_2_D_3_ by the cells of periodontal soft tissues, this conversion could enhance the innate immune defense in the oral cavity. Here we hypothesized that hGF and hPDLC have 1α-hydroxylase activity and can convert 25OHD_3_ to 1,25OH_2_D_3_. The objective of this study was to test the above hypothesis.

## Results

**Figure 1:**
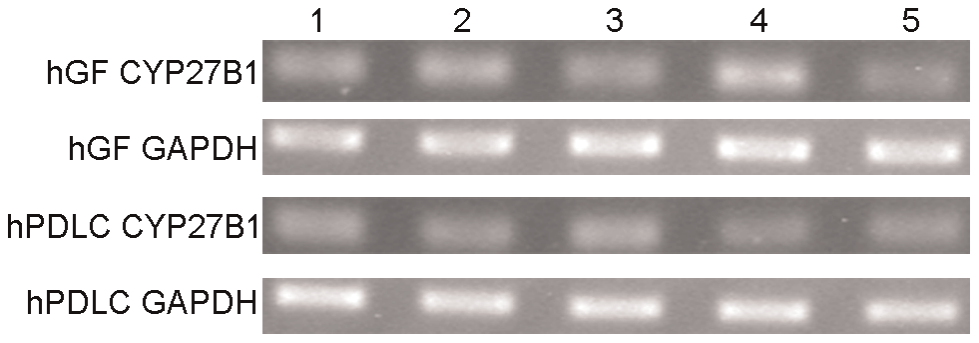
mRNA expression of CYP27B1 in hGF and hPDLC. mRNA expression of CYP27B1 was detected by RT-PCR in hGF and hPDLC from all five donors (each lane represents one donor). GAPDH was used as an internal control.

**Figure 2:**
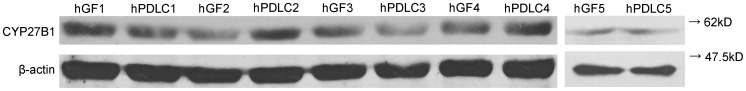
Protein expression of CYP27B1 in hGF and hPDLC. Protein expression of CYP27B1 was detected by Western blot in hGF and hPDLC from all five donors (donors are numbered 1–5). β-actin was used as an internal control.

1α-hydroxylase mRNA was detected in all the cells of the five donors (Fig. 1 and 2). The 1α-hydroxylase protein was detected with an antibody that detects 1α-hydroxylase expression in human osteoblasts, indicating that the 1α-hydroxylase in hGF and hPDLC could be the same CYP27B1 enzyme as that found in osteoblasts [15], [16].

**Figure 3:**
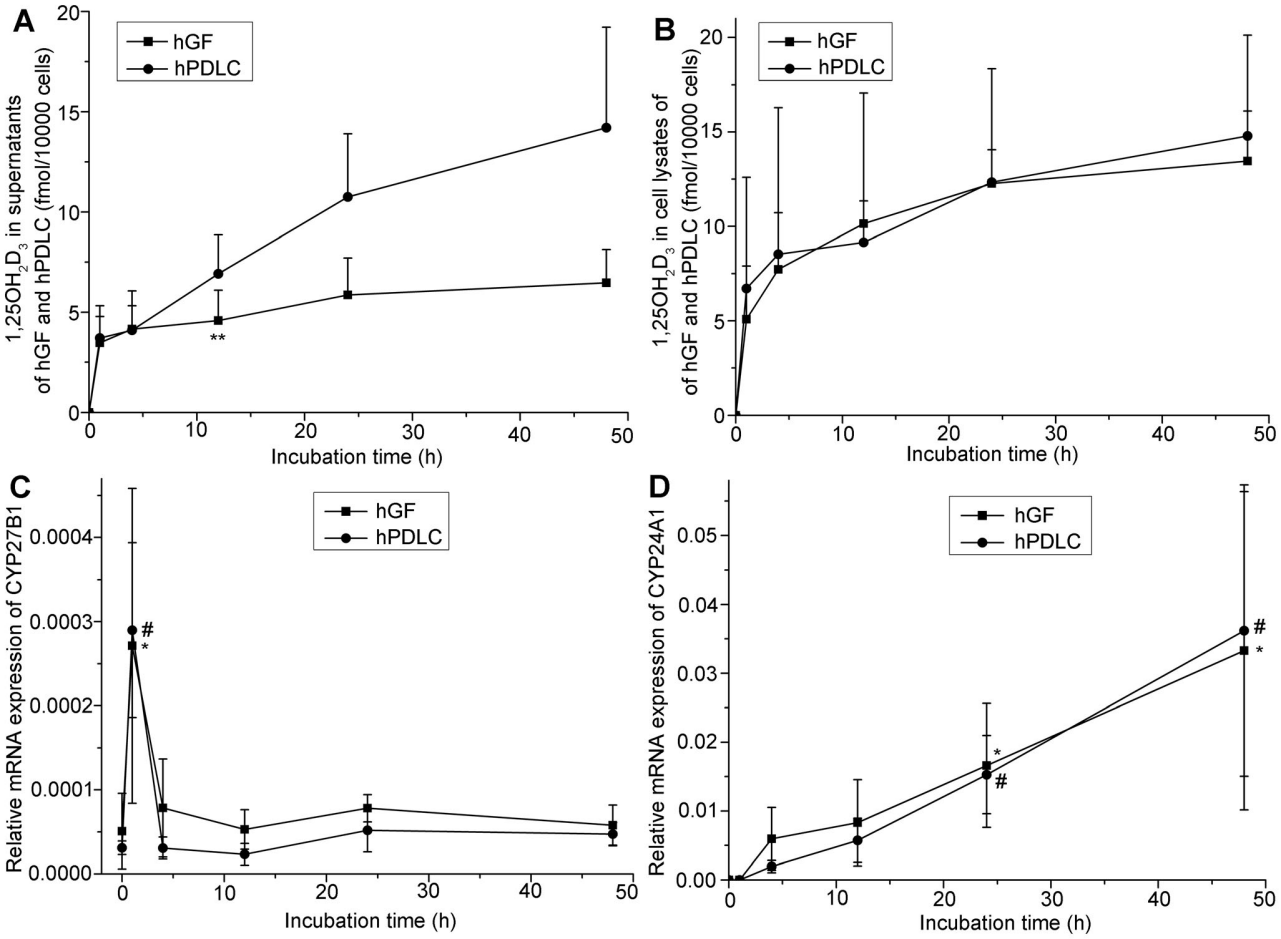
Activity of 1α-hydroxylase in hGF and hPDLC. hGF and hPDLC from donors 2, 3 and 5 were incubated with 1000 nM 25OHD_3_ for the times indicated and the production of 1,25OH_2_D_3_ was determined in (A) supernatants and (B) cell lysates. After prolonged incubation, production of 1,25OH_2_D_3_ increased. hPDLC released more 1,25OH_2_D_3_ than hGF 12 h after incubation with 25OHD_3_. The data is presented as the mean±SE. ** hGF generated significantly less 1,25OH_2_D_3_ than hPDLC at the same time point (*p*<0.05). The time course of CYP27B1 (C) and CYP24A1 (D) mRNA expression is also presented. CYP27B1 mRNA expression peaked at 1 h and no difference was detected between 0 h and any other time point. CYP24A1 mRNA expression was significantly higher at 24 h and 48 h than 0 h. * CYP27B1 or CYP24A1 mRNA expression at the time point was significantly different from that at 0 h in hGF (*p*<0.05). # CYP27B1 or CYP24A1 mRNA expression at the time point was significantly different from that at 0 h in hPDLC (*p*<0.05).

After confirming the expression of 1α-hydroxylase in hGF and hPDLC, the function of 1α-hydroxylase was investigated. While 1000 nM 25OHD_3_ did not have a significant cytotoxic effect on any of the cells within 48 h. hGF and hPDLC generated 1,25OH_2_D_3_ in response to 25OHD_3_ and the amount of 1,25OH_2_D_3_ increased with incubation time (Fig. 3A, B). The fact that extra- and intracellular 1,25OH_2_D_3_ was generated in the presence of 25OHD_3_ provides the most convincing evidence of the existence of 1α-hydroxylase in hGF and hPDLC. Although the total amounts of 1,25OH_2_D_3_ synthesized by hGF and hPDLC were not significantly different, more 1,25OH_2_D_3_ was released by hPDLC 12 h after adding 25OHD_3_. At all other time points, there was no significant difference in the levels of intracellular and extracellular 1,25OH_2_D_3_ between the two cell types. Moreover, CYP27B1 mRNA expression peaked at 1 h and no difference was detected between 0 h and any other time point (Fig. 3C). CYP24A1 mRNA expression increased over time and was significant higher at 24 h and 48 h than at 0 h (Fig. 3D).

**Figure 4:**
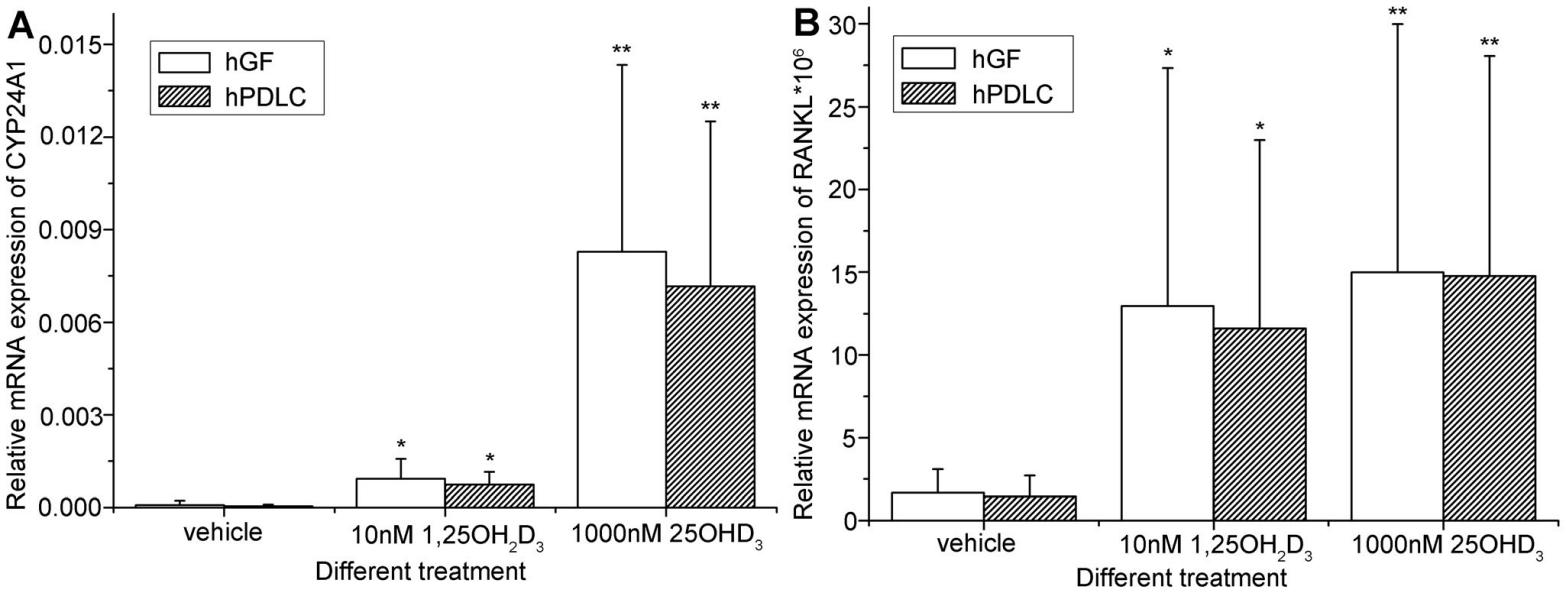
Effect of 25OHD_3_ incubation on gene expression in hGF and hPDLC. hGF and hPDLC from all five donors were treated with 1000 nM 25OHD_3_, 10 nM 1,25OH_2_D_3_ or vehicle for 48 h and mRNA expression of (A) CYP24A1 and (B) RANKL was examined by real-time PCR. The up-regulation observed after 1000 nM 25OHD_3_ treatment was significantly stronger than that observed after 10 nM 1,25OH_2_D_3_ treatment. The data are presented as the mean±SE. * denotes difference from vehicle (*p*<0.05). ** denotes difference from vehicle and 1,25OH_2_D_3_ (*p*<0.05).

In addition, exposure to 25OHD_3_ also resulted in an up-regulation of the 1,25OH_2_D_3_ responsive genes CYP24A1 and RANKL in hGF and hPDLC (Fig. 4A, B), which is further evidence of 1α-hydroxylase activity in hGF and hPDLC.

**Figure 5:**
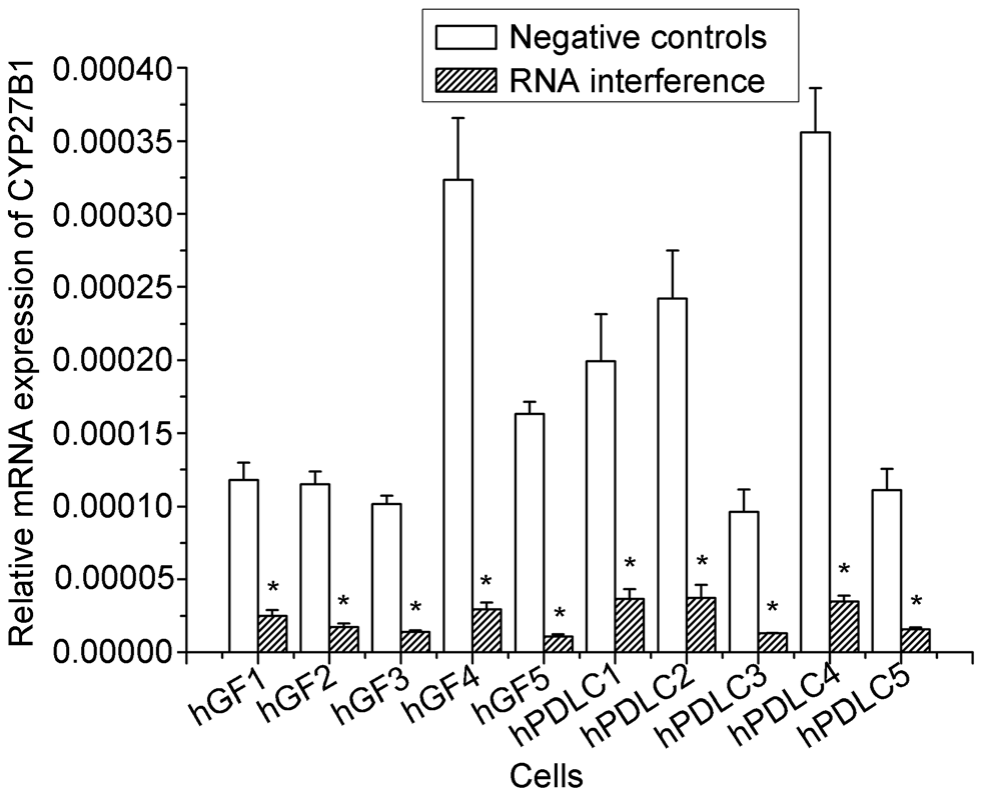
Efficiency of RNA interference against CYP27B1. All cells were transfected with either a siRNA oligonucleotide for CYP27B1 or a non-silencing control. Using real-time PCR as a measure, the efficiency of RNA interference against CYP27B1 was over 70% in hGF and hPDLC from all 5 donors. Donors are numbered 1–5. The data are presented as the mean±SD. * denotes difference from vehicle (*p*<0.05).

**Figure 6:**
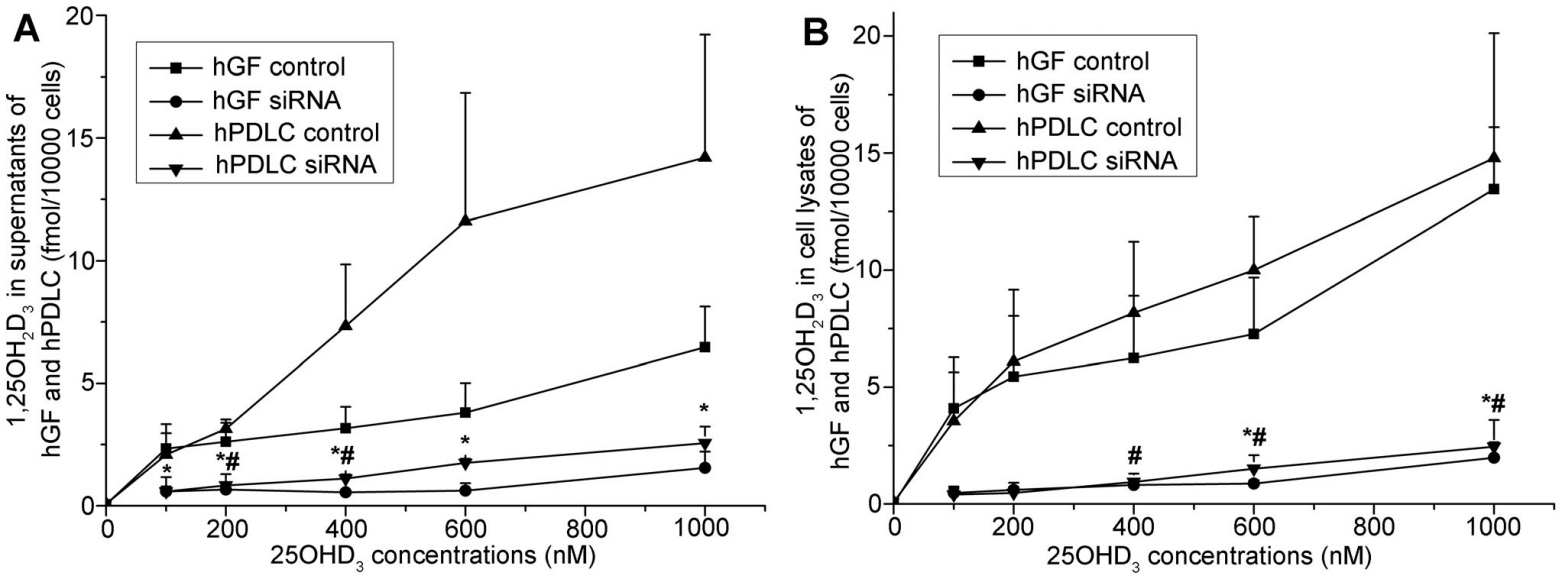
Effect of CYP27B1 silencing on 1,25OH_2_D_3_ generation. hGF and hPDLC from donors 2, 3 and 5 were treated with 25OHD_3_ at various concentrations indicated in the figure for 48 h after transfection with the siRNA oligonucleotide for CYP27B1 or a non-silencing control and 1,25OH_2_D_3_ production was measured in (A) supernatants and (B) cell lysates. When CYP27B1 was not silenced, production of 1,25OH_2_D_3_ increased with increasing concentration of 25OHD_3_. When CYP27B1 was silenced, the generation of 1,25OH_2_D_3_ decreased significantly compared with when CYP27B1 was not silenced. The data are presented as the mean±SE. * hGF generated significantly less 1,25OH_2_D_3_ with the same amount of 25OHD_3_ when CYP27B1 was knocked down (*p*<0.05). # hPDLC generated significantly less 1,25OH_2_D_3_ (with the same amount of added 25OHD_3_) when CYP27B1 was knocked down (*p*<0.05).

Based on this direct evidence for 1α-hydroxylase activity in hGF and hPDLC, we examined the effect of 1α-hydroxylase knockdown. The efficiency of RNA interference against CYP27B1 was over 70% (Fig. 5). The generation of 1,25OH_2_D_3_ increased with increasing 25OHD_3_ concentrations but dropped significantly when CYP27B1 was knocked down using specific siRNA (Fig. 6A, B).

**Figure 7:**
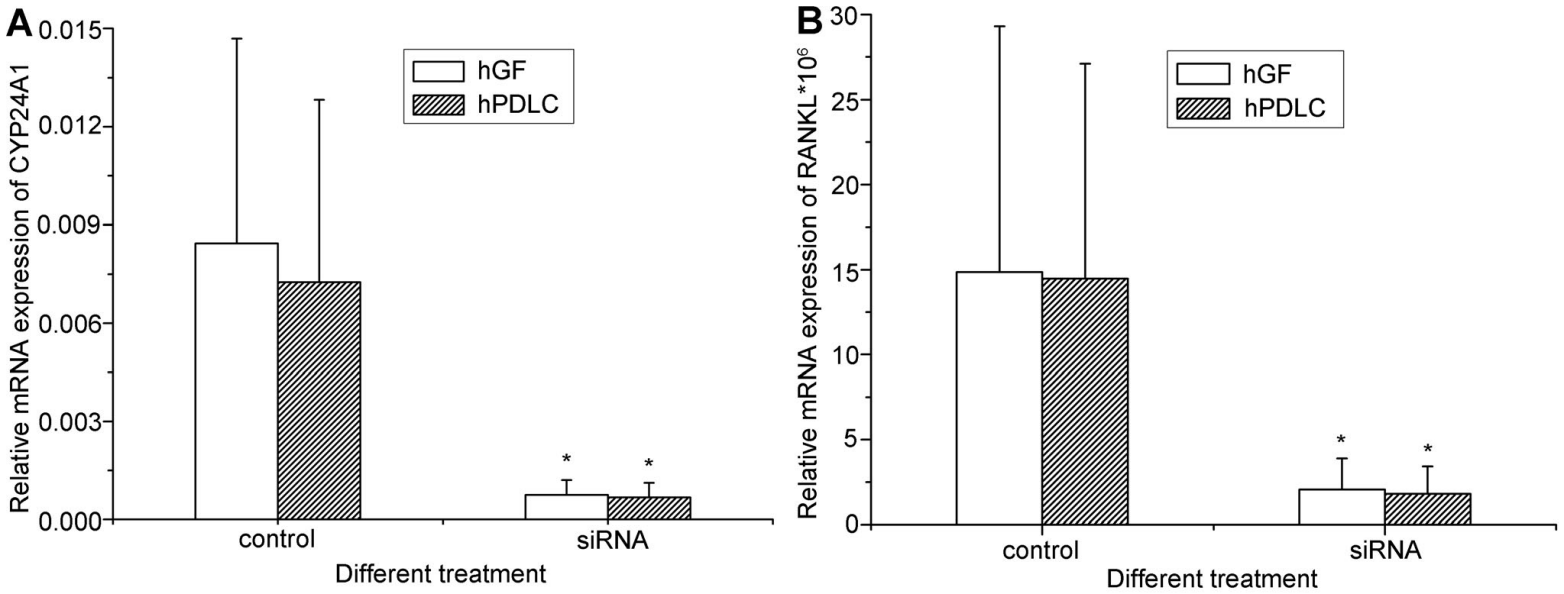
Effect of CYP27B1 silencing on gene up-regulation by 25OHD_3_. hGF and hPDLC from all five donors were treated with 1000 nM 25OHD_3_ for 48 h after transfection with the siRNA oligonucleotide for CYP27B1 or a non-silencing control and mRNA expression of (A) CYP24A1 and (B) RANKL was determined by real-time PCR. Compared with the transfection with a non-silencing control, transfection with the siRNA oligonucleotide for CYP27B1 resulted in a significantly weaker up-regulation of CYP24A1 and RANKL in both hGF and hPDLC. The data are presented as the mean±SE. * denotes difference from control (*p*<0.05).

Although 1,25OH_2_D_3_ is the major ligand of VDR, 25OHD_3_ could bind VDR with lower affinity and have a biological effect [22]. To address this issue, we knocked down CYP27B1 and found that the expression of CYP24A1 and RANKL decreased significantly (Fig. 7A, B), which strongly suggests that the effect of 25OHD_3_ in hGF and hPDLC occurs after its conversion to 1,25OH_2_D_3_. This is additional evidence for the activity of 1α-hydroxylase in hGF and hPDLC.

**Figure 8:**
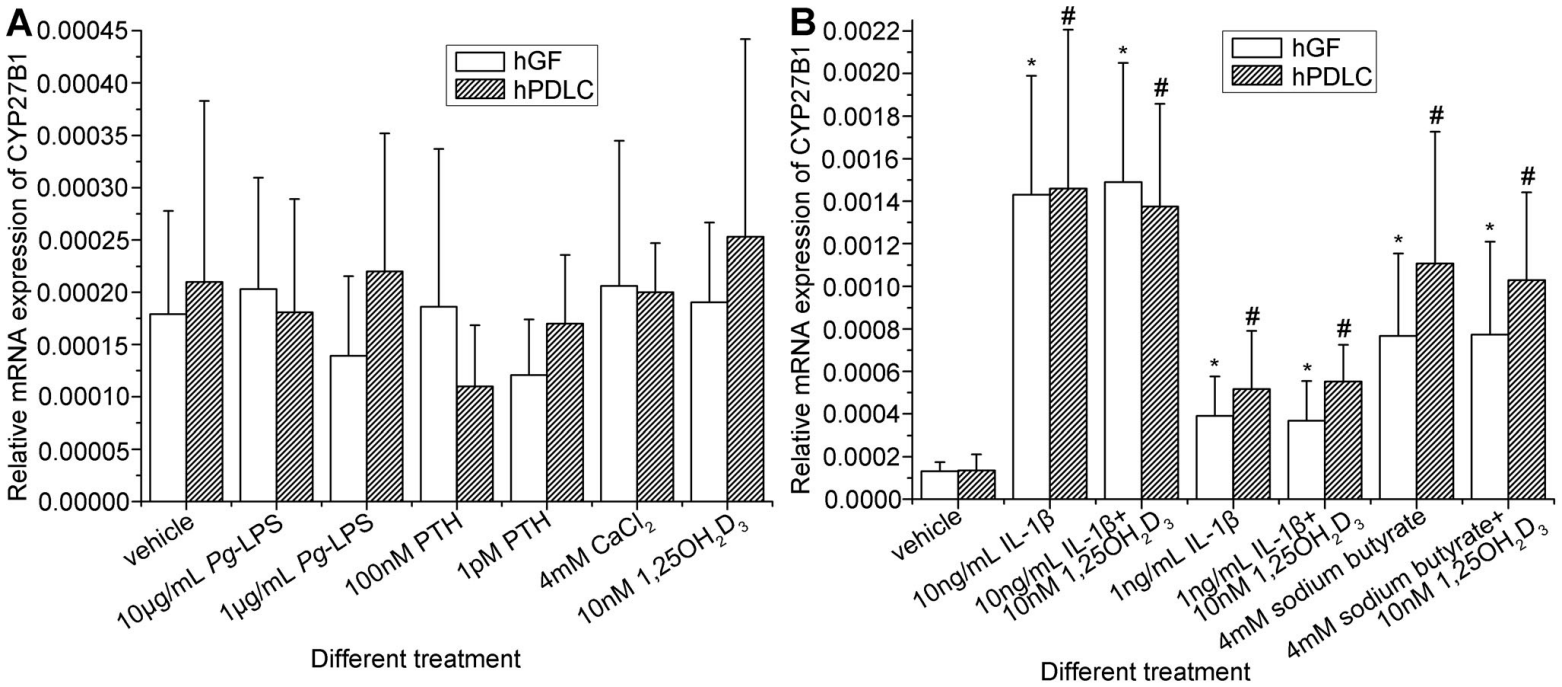
Preliminary investigation of CYP27B1 regulation in hGF and hPDLC. hGF and hPDLC from donors 2, 3, 4 and 5 were stimulated with different treatments indicated in the figure for 24 h and CYP27B1 expression was determined by real-time PCR. (A) *Pg*-LPS, parathyroid hormone, CaCl_2_ and 1,25OH_2_D_3_ did not significantly influence CYP27B1 mRNA expression. (B) IL-1β and sodium butyrate significantly up-regulated CYP27B1 mRNA expression independently of 1,25OH_2_D_3_. Additionally, the characteristics of CYP27B1 regulation in hGF and hPDLC were not significantly different. The data are presented as the mean±SE. * CYP27B1 mRNA expression was significantly different from that of the vehicle group in hGF (*p*<0.05). # CYP27B1 mRNA expression was significantly different from that of the vehicle group in hPDLC (*p*<0.05). IL-1β: interleukin-1β. *Pg*-LPS: *Porphyromonas gingivalis* lipopolysaccharide. PTH: parathyroid hormone.

After a comprehensive confirmation of 1α-hydroxylase activity in hGF and hPDLC, the regulation of 1α-hydroxylase in hGF and hPDLC was investigated. Parathyroid hormone (PTH), calcium, 1,25OH_2_D_3_ and *Porphyromonas gingivalis* lipopolysaccharide (*Pg*-LPS) did not significantly influence CYP27B1 mRNA expression in hGF and hPDLC (Fig. 8A). In contrast, interleukin-1β (IL-1β) and sodium butyrate strongly induced CYP27B1 expression independently of 1,25OH_2_D_3_ (Fig. 8B). In addition, no significant differences in the regulation of CYP27B1 was observed between hGF and hPDLC.

**Figure 9:**
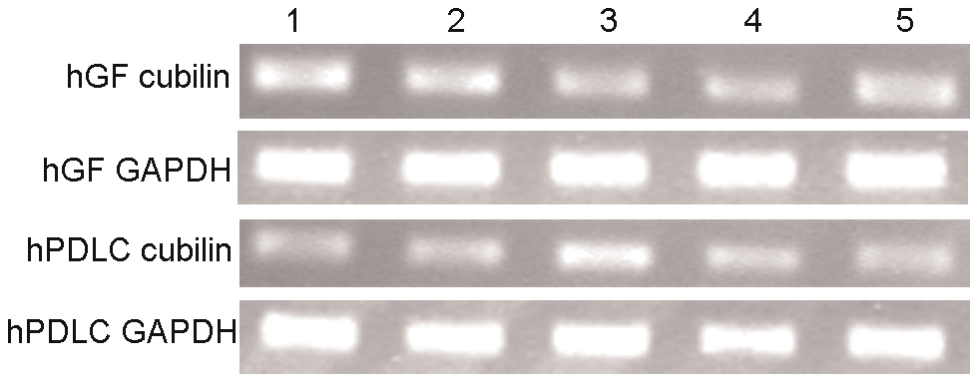
mRNA expression of cubilin in hGF and hPDLC. mRNA expression of cubilin was determined by RT-PCR in hGF and hPDLC from all five donors (each lane represents one donor). GAPDH was used as an internal control.

**Figure 10:**
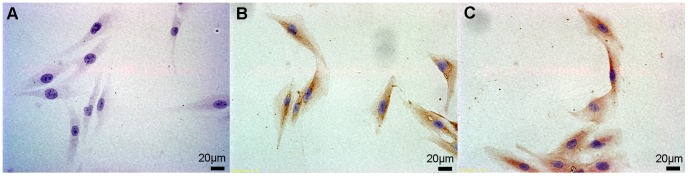
Protein expression of cubilin in hGF and hPDLC. hGF and hPDLC from all five donors were used for immunocytochemical staining of cubilin; expression of cubilin was detected in all the cells examined. Panel A is the negative control for the immunocytochemical staining of cubilin (400×). Panel B and C contain images of the hGF and hPDLC, respectively, from donor 2 (400×). The primary antibody was replaced with PBS for the negative control.

Despite the detection of the two kinds of DBP receptor expression, megalin mRNA expression was not detected. Both mRNA and protein expression of cubilin were detected in hGF and hPDLC (Fig. 9, 10).

## Discussion

Interestingly, the 1,25OH_2_D_3_ produced by hGF and hPDLC can influence vitamin D-responsive gene expression (Fig. 4, 7), an observation that provides a solid indication of an autocrine/paracrine action of vitamin D in periodontal fibroblasts. Because metabolism of 25OHD_3_ and 1,25OH_2_D_3_ by CYP24A1 is very important in the autocrine/paracrine action of vitamin D, we investigated the time course of CYP27B1 and CYP24A1 mRNA expression. CYP27B1 was only up-regulated at 1 h, while CYP24A1 mRNA expression increased over time (Fig. 3C, D). The results indicated that as more 1,25OH_2_D_3_ was synthesized, CYP24A1 was more robustly expressed, which resulted in more conversion of 1,25OH_2_D_3_ to 1,24,25OH_3_D_3_ and 25OHD_3_ to 24,25OH_2_D_3_, in turn resulting in less substrate for 1,25OH_2_D_3_ synthesis. Thus, hGF and hPDLC could regulate their own 1,25OH_2_D_3_ generation; this provided further evidence for autocrine/paracrine action of vitamin D.

Vitamin D binding protein (DBP) is the plasma carrier of vitamin D in humans [23]. Nykjaer et al. [24], [25] confirmed that vitamin D enters cells by receptor-mediated endocytosis and via the endocytic receptors megalin and cubilin. In the present study, cubilin mRNA and protein were detected in hGF and hPDLC (Fig. 9, 10), however, megalin was not detected despite the use of two pairs of primers. Atkins et al. [16] reported that osteosarcoma cell lines and primary osteoblast-like cells express abundant cubilin mRNA but only osteosarcoma cell lines express megalin mRNA. The cells used in our study were primary cells and their expression of DBP receptors was similar to the DBP receptor expression in primary osteoblast-like cells. Due to the presence of DBP receptors, plentiful 25OHD_3_ around periodontal fibroblasts [20] could be used as a substrate for 1,25OH_2_D_3_ synthesis. In addition, extracellular 1,25OH_2_D_3_ (Fig. 3A, 6A), generated by hGF and hPDLC, could enter the surrounding cells through cellular uptake associated with DBP, which suggests the possibility of paracrine action of vitamin D.

In the present study, CYP24A1 and RANKL mRNA expression were chosen to test the downstream biological effects of 25OHD_3_. It is striking that the biological effects caused by the locally produced 1,25OH_2_D_3_ after 1000 nM 25OHD_3_ treatment were much stronger than those achieved with exogenous 10 nM 1,25OH_2_D_3_ (Fig. 4A, B), and those similarly observed in treatment of osteoblasts [15]. After 1000 nM 25OHD_3_ treatment, 1,25OH_2_D_3_ concentration in the supernatants of hGF and hPDLC were 120 pM–360 pM and 120 pM–600 pM, respectively, much lower than 10 nM, but the biological effects were much stronger. One reason might be that after 25OHD_3_ treatment, 1,25OH_2_D_3_ is found not only in the supernatant but also in the cell lysates, allowing intracellular 1,25OH_2_D_3_ to bind VDR and influence the function of these cells directly. On the other hand, exogenous 1,25OH_2_D_3_ should enter the cells before eliciting a response. Thus, the direct availability at the site of action might be of great importance.

IL-1β in gingival crevicular fluids of patients with periodontitis reduced significantly after initial periodontal therapy, indicating that IL-1β is associated with periodontitis [20]. *Porphyromonas gingivalis* is an important pathogen of periodontitis and butyrate is its metabolite [26]. In our previous studies [27], [28], we demonstrated that the butyrate concentrations in gingival crevicular fluids of patients with periodontitis were significantly higher than those of healthy controls and butyrate levels in gingival crevicular fluids were significantly correlated with periodontal inflammation. To investigate the regulation of 1α-hydroxylase in hGF and hPDLC, IL-1β, *Pg*-LPS and sodium butyrate were chosen for the current study. However, although stimuli with periodontal characteristics were used here to simulate a periodontitis condition, this does not properly model a chronic disease situation in vivo and can only help to investigate the regulation of CYP27B1 in hGF and hPDLC. We found the NF-κB activator IL-1β to be a potent up-regulator of CYP27B1 mRNA in hGF and hPDLC (Fig. 8), which is in line with the observation in osteoblasts [15]. We also found that sodium butyrate could significantly up-regulate the expression of CYP27B1 while *Pg*-LPS could not, an observation which requires further study. Classical renal regulators of 1α-hydroxylase are parathyroid hormone, calcium and 1,25OH_2_D_3_ [10], [29]. Renal 1,25OH_2_D_3_ synthesis is primarily stimulated by low serum calcium, and consequently by parathyroid hormone that up-regulates CYP27B1 expression while 1,25OH_2_D_3_ itself down-regulates CYP27B1 by negative feedback [30]. In contrast to this, we found that 1α-hydroxylase expression in hGF and hPDLC was less sensitive to a regulation by these agents (Fig. 8). In addition, 1,25OH_2_D_3_ did not significantly change the up-regulation of CYP27B1 by IL-1β and sodium butyrate. A possible explanation might be that the induction of 1α-hydroxylase in hGF and hPDLC involves NF-κB pathways that are different from those involved in renal, cAMP-mediated protein kinase A signaling [31], [32] and negative vitamin D response element [33], [34]. This concept is similar to that of Hewison [35].

In the present study, each donor supplied both hGF and hPDLC and the CYP27B1 activity of hGF and hPDLC was compared. We demonstrated that hPDLC released more 1,25OH_2_D_3_ than hGF 12 h after adding 25OHD_3_. In the study of van D M et al. [15], osteoblasts incubated with 1000 nM 25OHD_3_ also generated 1,25OH_2_D_3_ and the concentration of 1,25OH_2_D_3_ in the supernatant was about 400–800 pM. In the present study, 1,25OH_2_D_3_ concentrations in the supernatants of hGF and hPDLC were 120 pM–360 pM and 120 pM–600 pM, respectively. Thus, osteoblasts might release more 1,25OH_2_D_3_ than hGF and hPDLC. hGF and hPDLC are two different types of fibroblasts of periodontal soft tissues. It has previously been demonstrated that hPDLC has a higher degree of similarity with osteoblasts than hGF [36], [37], [38]. This might be the reason for the higher release of 1,25OH_2_D_3_ by hPDLC than by hGF.

In recent years, increasing attention has been paid to the relationship between vitamin D and periodontitis. Dietrich et al. [39] reported that serum 25OHD_3_ concentrations are significantly and inversely associated with attachment loss in people aged over 50 years, however, there is no significant association between serum 25OHD_3_ concentrations and attachment loss in patients younger than 50 years. Dietrich et al. [40] also reported that subjects with high serum 25OHD_3_ concentrations were less likely to have bleeding on probing compared to subjects with low serum 25OHD_3_ concentrations. However, periodontally healthy subjects were not distinguishable from patients with either aggressive periodontitis or chronic periodontitis, and local 25OHD_3_ levels in gingival crevicular fluids were not detected in these studies. Thus, we designed our previous studies and found that patients with aggressive periodontitis had higher plasma 25OHD_3_ concentrations than healthy controls and that after periodontal therapy the higher plasma 25OHD_3_ concentrations were significantly reduced, indicating that 25OHD_3_ might be involved in periodontal inflammation [19], [20]. In addition, local 25OHD_3_ levels in gingival crevicular fluids were 300 times higher than plasma levels in patients with aggressive periodontitis [20]. Because 1,25OH_2_D_3_ may enhance the antibacterial defense of human gingival epithelial cells [21], the confirmation of 1α-hydroxylase activity in hGF and hPDLC implies that local 25OHD_3_ in gingival crevicular fluids might be metabolized to 1,25OH_2_D_3_ by hGF and hPDLC and be involved in the innate immune defense in the oral cavity. From this perspective, 1α-hydroxylase activity in hGF and hPDLC may benefit periodontal health. Recently, it was reported that calcium and vitamin D supplementation have a positive effect on periodontal health [41], [42]. However, topical application of vitamin D has not been reported. Since hGF and hPDLC have the ability to synthesize 1,25OH_2_D_3_, a topical application of 25OHD_3_ might fulfill the function of 1,25OH_2_D_3_. Thus, our data suggest a potential benefit of topical application of 25OHD_3_ in periodontal therapy.

Because no established cell lines of hGF or hPDLC are available, the cells used in the present study were all obtained from primary culture. Although all data were obtained from cell from at least 3 donors, the existence of individual differences among the cells from primary culture was the limitation of this study.

In conclusion, the results in the current study have identified for the first time hGF and hPDLC as new sites of extrarenal synthesis of 1,25OH_2_D_3_. Similar to other extrarenal 1α-hydroxylases, periodontal 1α-hydroxylase appears to fulfill an autocrine/paracrine function.

## Materials and Methods

### Ethics Statement

The study protocol was approved by the institutional review board of Peking University School and Hospital of Stomatology (PKUSSIRB-2011007) and written informed consent was obtained from each participant in accordance with the Declaration of Helsinki.

### Cell culture

Primary culture of hGF and hPDLC was carried out according to previously described methods [7], [26], [36], [43]. Briefly, hPDLC were prepared from extracted third molars of 5 young healthy volunteers and hGF were obtained from gingiva of the same 5 donors. The periodontal ligament tissues attached to the middle third of the roots were gently curetted by a surgical scalpel, minced and inoculated into 24-well plates. Gingiva from different donors was also minced and inoculated into 24-well plates. Tissue explants were maintained in Dulbecco's Modified Eagle's Medium (DMEM; Gibco, Grand Island, NY, USA) supplemented with 10% (v/v) fetal bovine serum (FBS; PAA, Coelbe, Germany), 100 U/ml penicillin G and 100 µg/ml streptomycin. Cultures were maintained in a humidified atmosphere of 5% (v/v) CO_2_ at 37°C. After reaching 80% confluence, hGF and hPDLC were digested with 0.25% (w/v) trypsin and 0.02% (w/v) EDTA and subcultured at a 1∶3 ratio.

DMEM without phenol red (Sigma, St. Louis, MO, USA), 10% dextran-coated charcoal stripped FBS (DCC-FBS; TBD, Tianjin, China) and cells of passage 4 were used in all of the following experiments. Experiments were all conducted in triplicate.

### Cytotoxicity test of 25OHD_3_

hGF and hPDLC from three donors were used in the cytotoxicity test. hGF and hPDLC in their logarithmic phase were plated into 96-well plates at a density of 3000 cells/well in DMEM with 10% DCC-FBS. 24 h later, the medium was replaced by DMEM without DCC-FBS. After another 24 h, the medium was changed to DMEM with 10% DCC-FBS and supplemented with 1000 nM 25OHD_3_ or vehicle, respectively. The cytotoxicity test was carried out according to the Cell Counting Kit-8 protocol (CCK-8; Dojindo, Kumamoto, Japan). Briefly, at hours 0, 24 and 48, cells were incubated with CCK-8 for the last 3 h of the culture period after which the optical density values (OD values) were determined at 490 nm with a microplate reader (Bio-Rad Model 550, Hercules, CA, USA).

### Detection of CYP27B1 expression

Cells from all five donor were seeded into 6-well plates at a density of 5000/cm^2^ in DMEM supplemented with 10% DCC-FBS. 4 days later, a portion of the cells were harvested using Trizol agent (Dongsheng Biotech, Guangzhou, China) for RT-PCR. RNA was extracted using Trizol according to the manufacturer's instructions and was reverse transcribed to cDNA using a reverse transcription kit (Bio-Rad, Hercules, CA, USA). PCR reactions were performed using the Taq PCR MasterMix (Tiangen Biotech, Beijing, China) in a Hybaid PCR Thermal Cycler (Thermo Scientific, Boston, Mass, USA). The primers used are listed in Table 1.

**Table 1:** Primer sequences used for PCR or real-time PCR.

Target genes	Forward primer (5′→3′)	Reverse primer (5′→3′)	Products (bp)
CYP27B1	GAAGTGCTAAGACTGTACCCTGT	CCTTGAAGTGGCATAGTGACAC	126
RANKL	GACATCCCATCTGGTTCCCA	CCCAACCCCGATCATGGTA	61
CYP24A1	GACTACCGCAAAGAAGGCTAC	CATCACTTCCCCTGGTTTCATTA	105
megalin	GCCGGCCAGTGGCCAAGAA	ACAGCGCAGCCAATTTCATCC	129
megalin	AAATTGAGCACAGCACCTTTGA	TCTGCTTTCCTGACTCGAATAATG	151
cubilin	TTCTTACGGGGTCTGCTCAAA	TGCAAGCCTTGGAATTTTCTCTC	226
GAPDH	GAAGGTGAAGGTCGGAGTC	GAAGATGGTGATGGGATTTC	226

The remaining cells were harvested using lysis buffer [20 mM Tris (pH 7.4), 150 mM NaCl, 1 mM EDTA, 1 mM EGTA, 1% (v/v) Triton X-100, 2.5 mM sodium pyrophosphate, 1 mM β-glycerol phosphate and 2 mM Na_3_VO_4_ supplemented with protease inhibitor cocktail (Roche, Mannheim, Germany)] [43] for Western blotting. Protein concentration was determined using the Bicinchoninic Acid Protein Assay Kit (Applygen, Beijing, China). 20 µg of total protein from each sample was loaded onto a gel comprising a 5% (w/v) stacking gel and a 10% (w/v) running gel. At the end of the electrophoresis, samples were transferred onto nitrocellulose blotting membranes (Hybond™; Amersham Pharmacia, Little Chalfont, UK). Blots were probed with a sheep polyclonal antibody to CYP27B1 (diluted 1∶200; The Binding Site Ltd., Birmingham, UK) or a mouse monoclonal antibody to β-actin (diluted 1∶1000; Santa Cruz Biotechnology, Santa Cruz, CA, USA). After washing, blots were incubated with horseradish peroxidase-linked secondary antibody. The secondary antibody against sheep (Kirkegaard & Perry Laboratories, Inc., Maryland, USA) and mouse (Beijing Zhongshan Golden Bridge Biotechnology, Beijing, China) were both diluted 1∶2500. Antigen-antibody complexes were detected using the Enhanced Chemiluminescence reagent (Applygen, Beijing, China).

### Measurement of 1,25OH_2_D_3_ production

Cells from 3 donors were treated with 1000 nM 25OHD_3_ (Sigma, St. Louis, MO, USA) for 1 h, 4 h, 12 h, 24 h or 48 h, after which supernatants were collected and a portion of cells were scraped in PBS containing 0.2% Triton X-100 and stored at −80°C. Prior to use, cell lysates were sonicated on ice in a sonifier cell disrupter for 2×15 s. 1,25OH_2_D_3_ levels in cell supernatants and cell lysates were detected using a 1,25OH_2_D_3_ radioimmunoassay kit (DiaSorin, Stillwater, MN) according to the manufacturer's instructions. The cross-reactivity with 25OHD_3_ was less than 0.01%.

In addition, a portion of the cells were harvested using Trizol reagent for the detection of the time course of CYP27B1 and CYP24A1 mRNA expression using real-time PCR. Real-time PCR reactions were performed using SYBR® Premix Ex Taq™ II (TaKaRa Biotechnology, Dalian, China) in an ABI 7500 real-time Thermocycler (Applied Biosystems, Foster city, CA, USA). The primers used are listed in Table 1. The data were analyzed using the SDS software according to the manufacturer's instructions. Glyceraldehyde-3-phosphate-dehydrogenase (GAPDH) was used as an internal control. The data are presented as relative mRNA levels calculated by the equation 2^−ΔCt^ (ΔCt = Ct of target gene minus Ct of GAPDH) [44].

### Detection of CYP24A1 and RANKL mRNA expression

Cells from five donors were incubated with 10 nM 1,25OH_2_D_3_ (Sigma, St. Louis, MO, USA), 1000 nM 25OHD_3_ or vehicle for 48 h before harvesting. Then RNA and cDNA were obtained and real-time PCR reactions were performed as described previously.

### RNA interference of CYP27B1

To confirm the dependence of 25OHD_3_ conversion into 1,25OH_2_D_3_ on CYP27B1, the highly specific technique of RNA interference was utilized. Cells were seeded at a density of 15000/cm^2^ in 6-well plates. 8 h later, cells were transfected with either CYP27B1 siRNA (10 nM) or non-silencing control siRNA using Hiperfect™ transfection reagent (Qiagen, Duesseldorf, Germany) according to the manufacturer's instructions. The target sequence of CYP27B1 siRNA was 5′-CTGGTTTACGGTTTCTTATAA-3′ and the non-silencing control was a non-homologous, scrambled sequence equivalent.

60 h after transfection, cells were harvested to confirm the effect of RNAi using real-time PCR. RNA and cDNA were obtained and real-time PCR was performed as described earlier.

After verifying the effect of RNAi, 1,25OH_2_D_3_ production after RNAi was determined. Cells were first transfected with CYP27B1 siRNA (10 nM) or non-silencing control siRNA and 12 h after transfection, these cells were treated with 200 nM, 400 nM, 600 nM or 1000 nM 25OHD_3_ (Sigma, St. Louis, MO, USA) for another 48 h. Then, the 1,25OH_2_D_3_ concentrations in the cell supernatants and cell lysates were determined as described earlier.

CYP24A1 and RANKL mRNA expression was also examined after RNAi. Cells were first transfected with CYP27B1 siRNA (10 nM) or non-silencing control siRNA and 12 h after transfection, these cells were treated with 1000 nM 25OHD_3_ for another 48 h. Then, CYP24A1 and RANKL mRNA expression was determined by real-time PCR.

### Regulation of CYP27B1 in hGF and hPDLC

A portion of the cells from four donors were incubated with IL-1β (PeproTech, London, UK; 1 ng/mL and 10 ng/mL), parathyroid hormone (PTH; SinoBio, Shanghai, China; 100 nM and 1 pM), *Pg*-LPS (Invivogen, San Diego, CA, USA; 1 µg/mL and 10 µg/mL), sodium butyrate (SCRC, Shanghai, China; 4 mM), CaCl_2_ (SCRC, Shanghai, China; 4 mM) and 1,25OH_2_D_3_ (10 nM) for 24 h. The other portion of the donor cells were incubated with IL-1β (1 ng/mL or 10 ng/mL) and 1,25OH_2_D_3_ (10 nM) or sodium butyrate (4 mM) and 1,25OH_2_D_3_ (10 nM) for 24 h.

### Detection of vitamin D binding protein receptor expression

The mRNA expression of megalin and cubilin were detected by RT-PCR as described earlier and the primers used are listed in Table 1.

Protein expression of cubilin was detected by immunocytochemistry. Cells from five donors were seeded on glass slides at a density of 20000/cm^2^ and 8 h later cells on glass slides were incubated with primary antibodies against cubilin (diluted 1∶50; Santa Cruz Biotechnology, Santa Cruz, CA, USA). The PV-9000 Polymer Detection System (Zhongshan Golden Bridge Biotechnology, Beijing, China) was used for immunocytochemical staining and a diaminobenzidine (DAB) kit (Zhongshan Golden Bridge Biotechnology, Beijing, China) was used to develop the colour followed by haematoxylin staining. The primary antibody was replaced by PBS for negative controls.

### Statistical Methods

The Shapiro-Wilk test was used to determinate the distribution of the variants. The Wilcoxon test and Friedman test were used to compare differences between the mRNA expression levels of CYP24A1 and RANKL in different groups. The effect of RNA interference and the effect of 25OHD_3_ exposure on CYP27B1 mRNA expression was analyzed using the paired samples t-test. Comparison of 1,25OH_2_D_3_ generation by hGF and hPDLC, comparison of 1,25OH_2_D_3_ generation with and without knockdown of CYP27B1 and comparison of CYP27B1 up-regulation in hGF and hPDLC were all performed using a paired samples t-test.

Statistical analyses were carried out using the SPSS 11.5 software package (SPSS Inc., Chicago, IL, USA). A *p* value<0.05 was considered statistically significant.

## References

[1] Rachez C, Freedman LP (2000). Mechanisms of gene regulation by vitamin D(3) receptor: a network of coactivator interactions.. Gene.

[2] von EM, Kongsbak M, Schjerling P, Olgaard K, Odum N (2010). Vitamin D controls T cell antigen receptor signaling and activation of human T cells.. Nat Immunol.

[3] Chen KS, DeLuca HF (1995). Cloning of the human 1 alpha,25-dihydroxyvitamin D-3 24-hydroxylase gene promoter and identification of two vitamin D-responsive elements.. Biochim Biophys Acta.

[4] Haussler MR, Whitfield GK, Haussler CA, Hsieh JC, Thompson PD (1998). The nuclear vitamin D receptor: biological and molecular regulatory properties revealed.. J Bone Miner Res.

[5] Christakos S, Dhawan P, Liu Y, Peng X, Porta A (2003). New insights into the mechanisms of vitamin D action.. J Cell Biochem.

[6] Kitazawa S, Kajimoto K, Kondo T, Kitazawa R (2003). Vitamin D3 supports osteoclastogenesis via functional vitamin D response element of human RANKL gene promoter.. J Cell Biochem.

[7] Tang X, Meng H (2009). Osteogenic induction and 1,25-dihydroxyvitamin D3 oppositely regulate the proliferation and expression of RANKL and the vitamin D receptor of human periodontal ligament cells.. Arch Oral Biol.

[8] Jones G, Strugnell SA, DeLuca HF (1998). Current understanding of the molecular actions of vitamin D. Physiol Rev.

[9] Takeyama K, Kitanaka S, Sato T, Kobori M, Yanagisawa J (1997). 25-Hydroxyvitamin D3 1alpha-hydroxylase and vitamin D synthesis.. Science.

[10] Bland R, Walker EA, Hughes SV, Stewart PM, Hewison M (1999). Constitutive expression of 25-hydroxyvitamin D3-1alpha-hydroxylase in a transformed human proximal tubule cell line: evidence for direct regulation of vitamin D metabolism by calcium.. Endocrinology.

[11] Zhang MY, Wang X, Wang JT, Compagnone NA, Mellon SH (2002). Dietary phosphorus transcriptionally regulates 25-hydroxyvitamin D-1alpha-hydroxylase gene expression in the proximal renal tubule.. Endocrinology.

[12] Lehmann B (1997). HaCaT cell line as a model system for vitamin D3 metabolism in human skin.. J Invest Dermatol.

[13] Khorchide M, Lechner D, Cross HS (2005). Epigenetic regulation of vitamin D hydroxylase expression and activity in normal and malignant human prostate cells.. J Steroid Biochem Mol Biol.

[14] Overbergh L, Stoffels K, Waer M, Verstuyf A, Bouillon R (2006). Immune regulation of 25-hydroxyvitamin D-1alpha-hydroxylase in human monocytic THP1 cells: mechanisms of interferon-gamma-mediated induction.. J Clin Endocrinol Metab.

[15] van DM, Koedam M, Buurman CJ, Hewison M, Chiba H (2006). Evidence for auto/paracrine actions of vitamin D in bone: 1alpha-hydroxylase expression and activity in human bone cells.. FASEB J.

[16] Atkins GJ, Anderson PH, Findlay DM, Welldon KJ, Vincent C (2007). Metabolism of vitamin D3 in human osteoblasts: evidence for autocrine and paracrine activities of 1 alpha,25-dihydroxyvitamin D3.. Bone.

[17] Kramer C, Seltmann H, Seifert M, Tilgen W, Zouboulis CC (2009). Characterization of the vitamin D endocrine system in human sebocytes in vitro.. J Steroid Biochem Mol Biol.

[18] Huang DC, Papavasiliou V, Rhim JS, Horst RL, Kremer R (2002). Targeted disruption of the 25-hydroxyvitamin D3 1alpha-hydroxylase gene in ras-transformed keratinocytes demonstrates that locally produced 1alpha,25-dihydroxyvitamin D3 suppresses growth and induces differentiation in an autocrine fashion.. Mol Cancer Res.

[19] Liu K, Meng H, Tang X, Xu L, Zhang L (2009). Elevated plasma calcifediol is associated with aggressive periodontitis.. J Periodontol.

[20] Liu K, Meng H, Lu R, Xu L, Zhang L (2010). Initial periodontal therapy reduced systemic and local 25-hydroxy vitamin D(3) and interleukin-1beta in patients with aggressive periodontitis.. J Periodontol.

[21] McMahon L, Schwartz K, Yilmaz O, Brown E, Ryan LK (2011). Vitamin D-mediated induction of innate immunity in gingival epithelial cells.. Infect Immun.

[22] Bouillon R, Okamura WH, Norman AW (1995). Structure-function relationships in the vitamin D endocrine system.. Endocr Rev.

[23] Haddad JG (1995). Plasma vitamin D-binding protein (Gc-globulin): multiple tasks.. J Steroid Biochem Mol Biol.

[24] Nykjaer A, Dragun D, Walther D, Vorum H, Jacobsen C (1999). An endocytic pathway essential for renal uptake and activation of the steroid 25-(OH) vitamin D3.. Cell.

[25] Nykjaer A, Fyfe JC, Kozyraki R, Leheste JR, Jacobsen C (2001). Cubilin dysfunction causes abnormal metabolism of the steroid hormone 25(OH) vitamin D(3).. Proc Natl Acad Sci U S A.

[26] Kurita-Ochiai T, Ochiai K, Suzuki N, Otsuka K, Fukushima K (2002). Human gingival fibroblasts rescue butyric acid-induced T-cell apoptosis.. Infect Immun.

[27] Li QQ, Meng HX, Gao XJ, Wang ZH (2005). Analysis of volatile fatty acids in gingival crevicular fluid of patients with chronic periodontitis.. Zhonghua Kou Qiang Yi Xue Za Zhi.

[28] Lu RF, Meng HX, Gao XJ, Feng L, Xu L (2008). Analysis of short chain fatty acids in gingival crevicular fluid of patients with aggressive periodontitis.. Zhonghua Kou Qiang Yi Xue Za Zhi.

[29] Bland R, Zehnder D, Hughes SV, Ronco PM, Stewart PM (2001). Regulation of vitamin D-1alpha-hydroxylase in a human cortical collecting duct cell line.. Kidney Int.

[30] Cross HS (2007). Extrarenal vitamin D hydroxylase expression and activity in normal and malignant cells: modification of expression by epigenetic mechanisms and dietary substances.. Nutr Rev.

[31] Rost CR, Bikle DD, Kaplan RA (1981). In vitro stimulation of 25-hydroxycholecalciferol 1 alpha-hydroxylation by parathyroid hormone in chick kidney slices: evidence for a role for adenosine 3′,5′-monophosphate.. Endocrinology.

[32] Henry HL (1985). Parathyroid hormone modulation of 25-hydroxyvitamin D3 metabolism by cultured chick kidney cells is mimicked and enhanced by forskolin.. Endocrinology.

[33] Murayama A, Kim MS, Yanagisawa J, Takeyama K, Kato S (2004). Transrepression by a liganded nuclear receptor via a bHLH activator through co-regulator switching.. EMBO J.

[34] Fujiki R, Kim MS, Sasaki Y, Yoshimura K, Kitagawa H (2005). Ligand-induced transrepression by VDR through association of WSTF with acetylated histones.. EMBO J.

[35] Hewison M, Zehnder D, Bland R, Stewart PM (2000). 1alpha-Hydroxylase and the action of vitamin D. J Mol Endocrinol.

[36] Somerman MJ, Archer SY, Imm GR, Foster RA (1988). A comparative study of human periodontal ligament cells and gingival fibroblasts in vitro.. J Dent Res.

[37] Ivanovski S, Li H, Haase HR, Bartold PM (2001). Expression of bone associated macromolecules by gingival and periodontal ligament fibroblasts.. J Periodontal Res.

[38] Xu Y, Jiang Y (2006). Mineralization effects of human platelet-rich plasma on human periodontium cells.. Zhonghua Kou Qiang Yi Xue Za Zhi.

[39] Dietrich T, Joshipura KJ, Dawson-Hughes B, Bischoff-Ferrari HA (2004). Association between serum concentrations of 25-hydroxyvitamin D3 and periodontal disease in the US population.. Am J Clin Nutr.

[40] Dietrich T, Nunn M, Dawson-Hughes B, Bischoff-Ferrari HA (2005). Association between serum concentrations of 25-hydroxyvitamin D and gingival inflammation.. Am J Clin Nutr.

[41] Garcia MN, Hildebolt CF, Miley DD, Dixon DA, Couture RA (2011). One-year effects of vitamin D and calcium supplementation on chronic periodontitis.. J Periodontol.

[42] Bashutski JD, Eber RM, Kinney JS, Benavides E, Maitra S (2011). The impact of vitamin D status on periodontal surgery outcomes.. J Dent Res.

[43] Tang X, Meng H, Han J, Zhang L, Hou J (2008). Up-regulation of estrogen receptor-beta expression during osteogenic differentiation of human periodontal ligament cells.. J Periodontal Res.

[44] Livak KJ, Schmittgen TD (2001). Analysis of relative gene expression data using real-time quantitative PCR and the 2(−Delta Delta C(T)) Method.. Methods.

